# Combined Therapy with Anthracyclines and GnRH Analogues for Breast Cancer: Impact on Ischemic Heart Disease

**DOI:** 10.3390/jcm12216791

**Published:** 2023-10-27

**Authors:** Maria Bergami, Olivia Manfrini, Edina Cenko, Raffaele Bugiardini

**Affiliations:** Laboratory of Epidemiological and Clinical Cardiology, Department of Medical and Surgical Sciences (DIMEC), University of Bologna, 40138 Bologna, Italy; maria.bergami@unibo.it (M.B.); olivia.manfrini@unibo.it (O.M.); edina.cenko2@unibo.it (E.C.)

**Keywords:** GnRH analogues, breast cancer, cardiotoxicity, ischemic heart disease

## Abstract

The combination of classic chemotherapy agents like anthracyclines with novel targeted medications has had a positive impact on women’s survival from breast cancer. GnRH analogues are primarily employed to temporarily suppress ovarian function in premenopausal women with hormone-receptor-positive (HR+) breast cancer. Despite their benefits, the true degree of their collateral effects has been widely understudied, especially when it comes to ischemic heart disease. This review aims at summarizing the current state of the art on this issue, with particular focus on the risk for cardiotoxicity associated with the combined use of GnRH analogues and anthracyclines.

## 1. Introduction

Breast cancer is one of the most frequent malignancies among women worldwide. In the United States, 297,790 new cases of breast cancer are estimated to be diagnosed in 2023, with an yearly increase in incidence of 0.5% [1]. The likelihood of developing breast cancer increases with age: approximately 50% of women diagnosed with breast cancer are 62 years or younger and 9% are below 45 years of age [2,3]. Despite its frequency, in recent years there has been a drastic improvement in breast cancer related prognosis, with overall 5-year survival rates ranging from 75% in the years 1975–1977 to 91% in the years 2012–2018 [2]. The combination of classic chemotherapy agents like anthracyclines with novel targeted medications like those employed in the so-called endocrine therapy (ET) may have contributed to improved outcomes. ET is primarily used to reduce estrogen levels in premenopausal women with hormone-receptor-positive (HR+) breast cancer. Inhibition of estrogen signaling slows down or stops the growth of HR+ breast cancer cells and also may mitigate metastasis formation. Currently, ET consists of the following: (1) Ovarian function suppression (OFS), usually obtained using gonadotropin-releasing hormone (GnRH) analogues; (2) Selective estrogen receptor modulators or down-regulators (SERMs or SERDs, namely tamoxifen or fulvestrant); (3) Permanent or reversible steroidal inhibitors of aromatase (AIs, namely exemestane, anastrozole, and letrozole), or a combination of two or more of the above drugs. 

The use of GnRH analogues usually determines several symptoms that resemble those elicited by menopause. As menopause is known to influence rates of cardiovascular risk factors and ischemic heart disease, the use of this drug class could impact negatively on cardiovascular health and especially on the risk of developing ischemic heart disease. This raises concerns regarding the administration of GnRH analogues in association with anthracyclines, which are known to induce myocardial dysfunction. This review aims at enunciating the effects of GnRH analogues on myocardial ischemia with special focus on a possible synergistic effect with anthracyclines.

### 1.1. Guidelines 

According to the ESMO Guidelines for Early Breast Cancer, ET is the therapeutic option of choice in early Luminal A type or Luminal B breast cancer, alone or in combination with chemotherapy [4].

GnRH analogues are primarily employed to temporarily suppress ovarian function in premenopausal women with hormone-receptor-positive (HR+) breast cancer, especially in cases of severe disease burden [4]. In 2018, the NICE Guidelines released a document reviewing available evidence for use of OFS in addition with other ET therapies in premenopausal women with estrogen-positive breast cancer [5]. In this statement, the committee found evidence of a clinical benefit derived from the use of OFS in combination with tamoxifen on overall survival rates in patients with ER-positive invasive breast cancer. Further information was provided by the Association of Breast Surgery Guidelines, which also took into account existing trials comparing the use of OFS in combination with some Aromatase Inhibitors such as exemestane [6]. These guidelines concluded that OFS in combination with other ET therapies could be considered in younger, premenopausal or perimenopausal women thought eligible for adjuvant chemotherapy, with an overall treatment duration of 5 years. These recommendations, however, do present important limitations, as they are based on few Randomized Clinical Trials that provided low-quality evidence of both benefits and harms of treatment with GnRH analogues, especially in terms of cardiovascular health. 

### 1.2. Anthracycline-Based Chemotherapy, Endocrine Therapy, and Risk of Cardiovascular Toxicities

The use of anthracycline-based chemotherapy in breast cancer treatment has been associated with an increased risk of cardiovascular toxicities. GnRH analogues, on the other hand, are primarily used to induce OFS, which may indirectly impact cardiovascular health through changes in risk factors for ischemic heart disease. Still, they are generally considered to have a lower risk of cardiovascular toxicities compared with anthracycline-based chemotherapy. However, there are some important considerations related to cardiac health that should be taken into account when using GnRH analogues either alone or in combination with anthracyclines and that will be discussed in the following paragraphs. 

### 1.3. GnRH Analogues: Mechanistic Aspects, Effectiveness, and Reasons for Concern

#### 1.3.1. Mechanism of Action of GnRH

**Figure 1:**
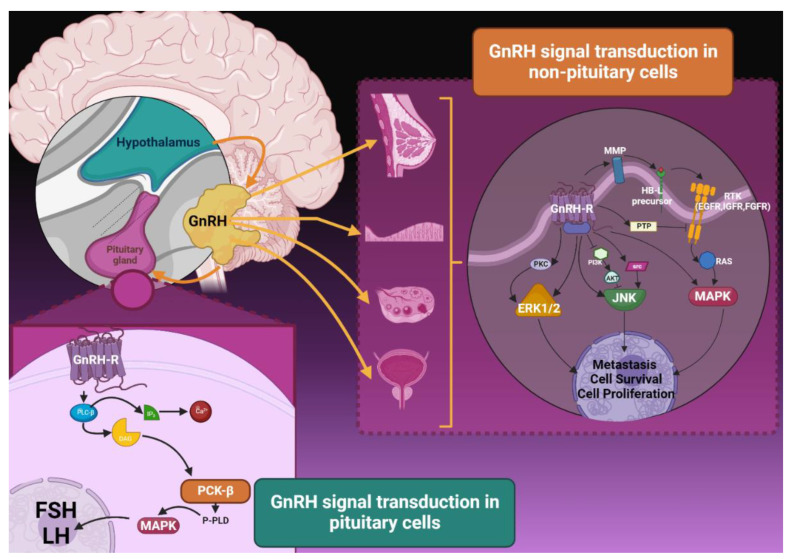
GnRH signal transduction pathways in pituitary and non-pituitary cells. Abbreviations: DAG, diacylglycerol; FSH, follicle-stimulating hormone; GnRH, gonadotropin-releasing hormone; GnRH-R, receptor for gonadotropin-releasing hormone; HB-L, HB ligand; IP3, Inositol 1,4,5-Trisphosphate; LH, Luteinizing hormone; MMP, matrix metalloproteinase; PLC-beta, phospholipase C beta; PCK-beta, protein kinase C beta type, PTP, protein tyrosine phosphatase; RTK, receptor tyrosine kinases; RAS, rat sarcoma virus. Image created on Biorender.com.

GnRH is a decapeptide produced in the hypothalamus that stimulates the peripheral release of luteinizing hormone (LH) and follicular stimulating hormone (FSH) by interacting with specific receptors localized on gonadotropin cells in the pituitary gland. LH and FSH have a final role in inducing the production and release of steroids in male and female gonads (Figure 1). GnRH is typically produced in a pulsatile manner: in women, estradiol and progesterone act as a negative-loop feedback and suppress the gonadotropin release in the luteal phase of the menstrual cycle [7]. 

The potential role of GnRH in the pathogenesis of malignancies of the reproductive tract, as well as its potential as a possible treatment pathway for such diseases, was first suggested by its detection in non-pituitary, both cancerous and non-cancerous tissues, i.e., ovaries, prostate, and myometrium. GnRH receptors have also been found in nearly 50% of breast cancer specimens, with a remarkable prevalence in TNBC, and invasive ductal carcinomas (prevalence of 64%), whereas their expression in normal breast tissues is yet to be proved [8,9].

GnRH-related signal transduction in non-pituitary cells presents several differences with the one described in the pituitary gland (Figure 1), even though both mechanisms mainly involve interaction with G-proteins. In pituitary cells the signaling cascade involves the increased activation of phospholipase C (PLC, Figure 1), which degrades into inositol phosphate (IP) and diacylglycerol (DAG). In turn, IP stimulates the release of Ca2+. The increase in DAG and Ca2+ levels induces the activation of Phosphokinase C (PKC) that triggers the synthesis and pulsatile release of gonadotropins via some members of the mitogen-activated protein kinase family (MAPK) [8,10]. In non-pituitary cells, two different forms of GnRH (GnRH I and II) have been detected, which manifest different physiopathological mechanisms according to the cellular context and have a direct impact on cellular growth, differentiation, and neoplastic potential. Their effects upon binding with GnRHR are determined by a process that acts independently from PLC, while still involving MAPK cascades like (ERK), Jun N-terminal kinase (JNK), and p38 MAPK. The effects of this signaling may favor or contrast apoptosis depending on the type of tissue involved and the duration of kinase signaling. They also influence the expression of metalloproteinases (MMP), which, along with Rho-GTPases and the urokinase-type plasminogen activator (uPA) system, induce cell mobilization and development of metastasis. In extra-pituitary tissues, GnRH and GnRHR are also involved in local angiogenesis via interaction with metabolism and functionality of the vascular endothelial growth factor (VEGF) and fibroblast growth factor (FGF), as well as by taking part in a cross-talk with receptor tyrosine kinases (RTK) like EGFR and insulin-like growth factors (Figure 1) [9]. 

#### 1.3.2. GnRH as a Treatment Target

The progressive unravelling of this complex variety of effects elicited by GnRH on extra-pituitary tissues has opened new promising roads for the treatment of several malignancies of the reproductive system, both in male and in female patients [8]. These therapeutic options involve the use of both GnRH agonists and antagonists, as they all exert a final suppressive effect on gonadotropin secretion, cellular growth, and metastasis formation in affected tissues, especially affecting hormone-dependent tumors [11,12,13]. The main difference between GnRH agonists and antagonists stand in treatment duration: the former need prolonged administration and higher dosages than the latter to determine an anti-neoplastic effect in hormone-dependent malignancies [8,9]. This is explained by understanding the mechanism of action of the two therapeutic agents: GnRH antagonists inhibit the secretion of gonadotropins and reduce sex steroid levels immediately after application by directly competing for receptor occupation [7,9]. Instead, GnRH agonists produce an initial transient increase in sex hormones, inhibiting FSH and LH synthesis only after prolonged and non-pulsatile administration of the drug has activated the negative-loop feedback signaling. 

#### 1.3.3. GnRH in Combination with Other Endocrine Therapies

As previously mentioned, these drugs have shown significant benefit in the overall and disease-free survival rates in several studies. Two large scale trials, Suppression of Ovarian Function Trial (SOFT) and Tamoxifen and Exemestane Trial (TEXT), analyzed the role of pharmacological or surgical OFS in addition to tamoxifen or exemestane on prognosis of female breast cancer patients [14]. Overall, in the SOFT trial there was a modest benefit of OFS added to tamoxifen vs. tamoxifen alone on overall survival at 8 years (93.3 versus 91.5 percent; HR 0.67, 95% CI 0.48–0.92), especially in younger women, while the same phenomenon was not observed in the exemestane group. The vast majority (94%) of participating women had also received adjuvant chemotherapy, and this subgroup was actually the one to exert the higher benefit from combined treatment (disease-free survival rates: 71.4% in the tamoxifen only arm vs. 76.7% for tamoxifen + OFS; HR 0.76 (0.6–0.97) vs. 80.4% for exemestane + OFS; HR 0.68 (0.53–0.88) in the SOFT trial. A large meta-analysis on 16 trials also concluded that GnRH agonists given in addition to tamoxifen, chemotherapy or both appeared to be beneficial both in terms of recurrence and of death after recurrence (relative risk reduction of 12.7% and 15.1% respectively) [15]. 

#### 1.3.4. GnRH and Anthracyclines: An Understudied Combination

The encouraging body of evidence competing GnRH analogues and breast cancer prognosis has led to the search and development of association drugs linking GnRH analogues with doxorubicin: AN-152 (also known as AEZS-108) proved to be effective in animal models and relatively safe to be tested in phase II and III trials [16,17]. Still, the sole phase III trial designed for its use in breast cancer patients was terminated early due to poor recruitment, so we have no information on its efficacy and toxicity in this population [18]. 

The issue with toxicity is of remarkable importance when it comes to anticancer treatment, especially when it comes to the effect of these medications on cardiovascular health. With regards to breast cancer, anthracyclines are by far the most renown cytotoxic agents with major life-limiting cardiotoxic effects, which can appear quite early into treatment [19]. These effects mainly lead to myocardial dysfunction, and do appear to frequently hinder the results obtained with chemotherapy, not only by reducing patients’ life expectancy but also by significantly worsening their quality of life. In turn, by observing women’s decrease in cardiovascular health following menopause, it is reasonable to suppose that GnRH analogues may too exert a cardiotoxic effect, even though on a longer run and by means of a different pathophysiology. Unfortunately, while this theme has recurred in several studied conducted on the male population, in women the body of evidence is scarce and limited. Still, it is possible to delineate a possible pattern of GnRH-related cardiotoxicity by taking into consideration the risk factors and culprit mechanisms leading to one of the main causes of myocardial dysfunction in the overall population, which is ischemic heart disease. Combining the damage induced by ischemia to the one cause by chemotherapy could, in fact, further worsen the prognosis and life of women affected by breast cancer and, thus, deserves our complete attention. 

## 2. GnRH Analogues and Risk Factors for Ischemic Heart Disease

### 2.1. Insulin Resistance and Diabetes

Fertility status in women is known to influence the likelihood of developing insulin resistance and diabetes, and this relation seems to be time-dependent. A recent meta-analysis gathering data from 191,762 postmenopausal women across thirteen studies observed that patients reaching menopause before 45 years of age (early menopause) and those with premature ovarian insufficiency all had a higher risk of developing diabetes than those entering their menopause later in life [20]. The potential impact of hormonal status in women on diabetes risk is warranted by two main randomized control trials, namely the Women’s Health Initiative Hormone Trial [21] and the Heart and Estrogen/progestin Replacement Study [22]. Both studies found that hormonal therapy with estrogen and progestin led to a statistically significant reduction in risk for diabetes in postmenopausal women. Furthermore, data from 83,799 French women from the E3N (Etude Épidémiologique de Femmes de la Mutuelle Générale de l’Education Nationale) cohort study showed that longer exposure to physiological sex hormones had an inversely proportional association with incidence of diabetes, whereas prolonged use of contraceptive pill increased the risk of developing the disease by 33% [23]. Higher estrogen levels may, thus, act as protective factors in preserving correct glucose metabolism: in animal models, estrogen receptors, ER alpha and ER beta, have been detected in pancreatic beta cells, and prolonged exposure to physiological levels of 17 beta-estradiol have shown to directly influence insulin production and release [24]. Although such a direct relationship between estrogen levels and insulin resistance was not completely confirmed in human subjects, with a possible dose-dependent effect of sex hormones on risk for diabetes having been observed in smaller studies, the overall evidence presented above justifies concern when considering ovarian suppression treatment by GnRH analogues in premenopausal women affected by breast cancer. Still, research in this field is scarce. In 2017, a study conducted by the Women’s Health Initiative concluded that, while hysterectomy did significantly increase the risk of developing diabetes, the same could not be said for bilateral oophorectomy, which is a surgical form of OFS currently considered a valid alternative to GnRH analogues [25]. The authors attributed this finding to the fact that OFS via oophorectomy led to a decrease in both estrogen and androgen levels; thus, the latter phenomenon may have counterbalanced the hyperglycemic activity of lower estrogen. However, it should be noted that GnRH agonists seemingly worsened glucose metabolism in male patients treated for prostate cancer (HR 1.44 for development of diabetes, *p* < 0.001) [26,27]. 

The potential synergistic action of GnRH analogues in combination with other forms of ET, such as taxane and exemestane, should also be taken into account. In the ECOG trial, patients administered with chemotherapy in combination with goserelin alone or goserelin and tamoxifen, showed higher rates of diabetes than with chemotherapy alone [28]. An adjunctive and enhancing action of GnRH analogues on a pro-diabetic effect of anthracyclines cannot be fully excluded. In fact, experimental pieces of evidence suggest a possible connection between doxorubicin use, imbalanced insulin signaling, and cardiac insulin resistance [29]. In a study conducted on a series of Wistar rats, it appeared that doxorubicin administration significantly increased levels of insulin, glucose and FFA within only 72 h from infusion. This was accompanied by a decreased expression of GLUT4 and AMPk α (pT172), which participate in the mechanism of peripheral glucose uptake [30]. 

Future research is warranted to shed light on this still-foggy association between GnRH analogues, chemotherapy, and insulin resistance. Development of diabetes mellitus is a strong predictor for adverse cardiovascular events in women, having a direct impact on both atherosclerosis progression [31] and microvascular dysfunction [32], and acting as a potential trigger for myocardial ischemia even in the absence of obstructive disease [33]. 

### 2.2. Lipid Metabolism and Dyslipidemia

The fact that serum estrogen levels do influence lipid metabolism has been documented before, but this association appears to be far from linear. Estrogen has been found to directly interact with the hepatic cells and act as a regulating factor in the process of fatty acids oxidation and very-low-density lipoprotein production (VLDL). In this regard, it has been shown that, when compared with men, premenopausal women do present lower concentrations of VLDL molecules, which, however, present a higher density than those found in their male counterparts [34]. This leads to a more rapid VLDL turnover, with an increased rate of reabsorption of this molecule from the bloodstream. This may lead to the belief that lower levels of estrogen should unequivocally mean higher levels of VLDL, lower turnover rates, and an overall worsening of lipid metabolic profile. However, this association cannot be confirmed with certainty based upon currently available evidence [35,36,37]. When comparing premenopausal and postmenopausal women, one would expect the latter to present a lipid profile much similar to that of men. Although there are small differences in terms of LDL and apolipoprotein B-100 concentrations that may be imputable to changes in hormone status throughout the course of life [38], several studies suggest that dyslipidemia in postmenopausal women is mainly attributable to change in body composition and insulin resistance. Similar observations can be made for women affected by polycystic ovary syndrome [39,40]. These conflicting results reflect the somewhat scarce and fragmented data we currently have on the effect of GnRH agonists on the risk of developing dyslipidemia. In fact, the available studies on this issue date back 30 years [41,42,43,44], do not involve breast cancer patients specifically, and show conflicting results. While the majority of them generally confirm an absence of lipid-related side effects in the treatment with GnRH analogues [45], an early prospective study found that GnRH analogues significantly increased total cholesterol (TC), high-density lipoprotein cholesterol (HDL-C), and triglycerides (TG) [46]. In a 2004 trial enrolling 100 premenopausal women with symptomatic uterine leiomyomas, the authors compared the metabolic effects of GnRH analogues alone or in combination with raloxifene, a selective estrogen receptor modulator [47]. In the group of patients treated only with GnRH analogues, follow-up levels of TC, HDL-C, LDL-C, and TG were significantly higher than in the group administered with raloxifene as an add-back treatment, even though the lipid profile had worsened in both treatment arms when compared to baseline. This, of course, remarks the importance of considering all therapeutic agents involved in treatment for breast cancer when investigating risks. Tamoxifen, for instance, has been shown to increase serum levels of HDL and lower those of LDL, thus counterbalancing potential adverse effects of GnRH analogues [48,49]. Instead, even though evidence is limited, anthracyclines have shown to be potentially detrimental for lipid metabolism. This association was initially observed in animal studies, where doxorubicin induced a three-fold increase in LDL levels 14 days after injection [50,51]. Anthracyclines have also appeared to be independently associated with higher levels of triglycerides in a retrospective analysis conducted on 1934 patients undergoing adjuvant chemotherapy [52]. 

The possibility of a synergic effect on lipid metabolism of GnRH analogues and anthracyclines is still to be investigated, but, if proved, this phenomenon would further remark the importance of an adequate lipid-lowering approach in breast cancer women undergoing treatment. Rosuvastatin has already been studied in combination with doxorubicin, and benefits both in terms of oxidative stress reduction and dyslipidemia control were detected [51]. Future trials should further expand on the issue by comparing different combined treatment strategies, including those with GNRH analogues.

### 2.3. Hypertension

In the ECOG trial [28], the addition of goserelin to cyclophosphamide and doxorubicin-based chemotherapy significantly increased the risk of hypertension, a phenomenon that seemed independent from the administration of tamoxifen.

In male patients undergoing treatment for prostate cancer, GnRH analogues have been associated with increased arterial stiffness, a phenomenon generally attributed to the fall of androgen’s protective influence on vasodilation [53]. Estrogen exerts a similar action, as suggested by the evidence of an acceleration in arterial stiffness one year following the final menstruation [54]. The mechanisms underlying the association between pharmacological or natural menopause and hypertension seem to be mainly related to a reduced production of vasodilators like nitric oxide and PGI2 induced by the fall in estrogen levels. Estrogen has also been shown to inhibit circulating renin and ACE and to downregulate ATi receptor both in the hypothalamus and in peripheral tissues [55]. Ovarian suppression increases the expression of ATi receptors, increasing blood pressure especially in salt-sensitive subjects. Considering the risk of cardiovascular disease associated with hypertension, especially if in association with other cardiovascular risk factors, active blood pressure monitoring and dietary salt restriction could be advisable preventive strategies in women initiating GnRH analogues for breast cancer treatment. 

### 2.4. Obesity and Weight Gain

An increase in fat deposits has been subject to widespread research, especially on its association with worsening cardiovascular health. Obesity has been shown to be related with an increased risk of ischemic heart disease [56]. Throughout the years, some authors have argued against the inclusion of overweight and obesity among the list of independent predictors for cardiovascular disease, as it appeared that metabolically healthy obese individuals, that is people with BMI over 30 and no other cardiovascular risk factor, did not show increased likelihood of ischemic heart disease if compared with their normal-weighted counterparts [57,58]. Still, there is a consistent body of research suggesting that an increase in body fat deposits, especially if visceral, do act as a direct driver for vascular damage and atherosclerosis [59]. In women, menopause comes with an increased risk of weight and, even more so, with a redistribution of body fat towards visceral and subcutaneous adipose tissue [60]. This change has been attributed to increased levels of testosterone after menopause rather than a decrease in estrogen [61]. 

An increase in body weight has been widely observed following a breast cancer diagnosis and it is correlated with a poor prognosis. Although the possibility of a direct association between breast cancer treatment and obesity has not been proven universally [62], the use of GnRH analogues has been described to induce weight gain in several studies [5,28]. As obesity and overweight have been demonstrated to be independently associated with a higher risk of cardiotoxicity derived from anthracycline treatment (pooled odds ratio 1.38 (95% CI, 1.06 to 1.80)), patients administered with GnRH analogues should be closely monitored for prevention and prompt management of weight gain.

## 3. GnRH Analogues and Cardiovascular Outcomes: Ischemic Heart Disease and Left Ventricular Dysfunction

The association between sex and ischemic heart disease is complex and multifaceted, and the need to better understand the social and biological factors involved in this relationship has gained increasing attention over the past years. Atherosclerosis, which is one of the main pathophysiological mechanisms underlying coronary artery disease and ischemic heart disease, appears to be less pronounced in women under 60 years of age when compared with men of similar age. Still, when considering the most severe form of myocardial infarction, ST-segment-elevation myocardial infarction (STEMI), there does not seem to be a significant difference in prevalence across sexes [63]. On the other hand, several studies report increased mortality following STEMI in younger, but not in older, women compared with men [64]. Although part of this difference could be explained by disparities in treatment and psychosocial factors, increasing evidence supports the existence of intrinsic, biological sex differences that could make STEMI particularly dangerous for younger women [65]. Although it would be reductive to attribute all of these biological features to the hormonal status of the patients, some aspects of the aforementioned discrepancies show connections with the effects of estrogen on peripheral tissues. One of said connections could be that of estrogen and microvascular dysfunction [66]. Indeed, experimental data on mice models suggest that estrogen may enhance the release of nitric oxide (NO) and prostaglandin (PG) at microvascular level, thus favoring both flow-dependent and pressure-dependent dilation of the arterioles. 

An effect of sex hormones on the development and damage of atherosclerotic plaques can also be hypothesized. In a recent study by Segeers et al., it was observed that, although an overall analysis men and women presented comparable distribution of luminal plaque morphology, sex differences appeared when stratifying the population by age [67]. Whereas the proportion between cases of plaque ruptures and plaque erosions appeared to be similar in men independently from age, women < 50 years had higher rates of plaque erosions than their older counterparts. Moreover, in female patients rates of plaque ruptures significantly increased with age, with a correspondent shift in plaque composition towards a thinner fibrous cap. This is in accordance with previous evidence showing that higher estrogen levels may exert an anti-inflammatory effect with regards to plaque rupture, but not plaque erosion. In fact, estrogen is associated with higher levels of myeloperoxidase, hyaluronan deposition, and CD 44 in activated smooth muscle cells. These molecules are also increased in eroded plaques [68]. It derives that a physiological or pharmacologically induced decrease in estrogen could lead to changes in plaque composition and in the epidemiology and pathophysiology of ischemic heart disease in women. By inhibiting the secretion of FSH and LH, GnRH analogues may on the one hand decrease the production of NO and PG, thus enhancing microvascular reactivity, and on the other they could alter plaque composition, making younger women at higher risk of plaque rupture. Additionally, GnRH analogues do not seem to influence the risk of platelet aggregation, although evidence in this direction is scarce: in a small study by Pinto et al., administration of buserelin did increase levels of thromboxane A2, but this effect was not followed by higher levels of thrombin or by an exacerbated tendency towards platelet aggregation [69]. 

Another element that suggests a potential impact on ischemic heart disease of GnRH analogues is the fact that vasomotor symptoms like hot flushes are to date the most common adverse effect of this drug. A large meta-analysis of 10 studies that included 213, 976 women reported that the presence of this symptom and other menopausal symptoms was associated with an increased risk of coronary heart disease (risk ratio 1.28, 95% CI 1.08–1.52) [70,71].

From an epidemiology standpoint, GnRH analogues have demonstrated the risk of coronary heart disease (adjusted HR, 1.16; *p* < 0.001), myocardial infarction (adjusted HR, 1.11; *p* = 0.03), and sudden cardiac death (adjusted HR, 1.16; *p* = 0.004) [26] in men treated for prostate cancer. However, despite the pathophysiological mechanisms mentioned above, in female patients evidence is scarce and controversial. An observational study conducted on 172,850 female patients with breast cancer found that use of GnRH agonists was associated with a reduced risk of ischemic heart disease (HR: 0.50; 95% CI: 0.39–0.64) [72]. However, this analysis did not take into account the concomitant or sequential administration of adjunctive medications that could have influenced outcomes, especially considering that the study comprised cases ranging from 2000 to 2018. As demonstrated in the ATLAS trial, use of tamoxifen is associated with a reduction in the risk of ischemic heart disease, even when administering the drug for up to 10 years [73]. This could potentially imply an attenuating effect on the pro-ischemic characteristics of GnRH analogues. In 2011, a randomized control trial from the SOFT investigators compared the incidence of outcomes and complications in premenopausal breast cancer patients undergoing treatment with tamoxifen alone, tamoxifen in combination with ovarian suppression (either pharmacological or surgical), or exemestane plus ovarian suppression. The results showed low rates of myocardial ischemia at a 5-year follow-up in both arms taking tamoxifen (0.1% combined therapy vs. 0.4% tamoxifen alone). As, however, use of ovarian suppression seemingly increased the incidence of most cardiovascular risk factors, the authors rightly concluded that longer follow-up would be needed to assess potential differences in cardiovascular outcomes [74]. 

Radiotherapy has too been linked to an increased risk of ischemic heart disease: in a case-control study conducted by Darby et al., it was observed that the rate of major coronary events increased by 7.4% for each adjunctive 1 Gy delivered to the heart (95% CI, 2.9 to 14.5; *p* < 0.001) [75]. The development of radiation-induced coronary disease can be explained by multiple mechanisms. Through the formation of free radicals, radiation causes an endothelial injury in the coronaries, thus ensuing a proinflammatory state that ultimately leads to ruptured vessel walls, platelet aggregation, thrombosis, and intima fibrosis. These mechanisms appear to be enhanced by the use of other anticancer medications like anthracyclines. However, a comparable effect was not observed when analyzing concomitant administration of radiotherapy and OFS [75].

Although there are no definite data on the role of anthracyclines in increasing risk for ischemic heart disease, and especially on their synergistic effects when used combination with GnRH analogues, the cardiotoxic effects of this drug class are renowned and well-documented. The main issue developed as a consequence of their use is hypokinetic cardiomyopathy, whose first description dates back to 1967 [76]. As defined by the 2022 ESC Guidelines on Cardio-oncology [19], hypokinetic cardiomyopathy is characterized by the manifestation of heart failure symptoms, either mild or severe, as well as a reduction in left ventricular ejection fraction, decline in global longitudinal strain, and/or rise in cardiac biomarkers. Patients treated with anthracyclines show consistently higher levels of cardiac troponins, which do further increase along with treatment prosecution. This association suggests that direct myocardial injury is involved in the pathogenesis of anthracyclines-induced cardiotoxicity. An increase in cardiovascular risk factors or a possible pro-ischemic effect of GnRH analogues in women affected by breast cancer could exacerbate the likelihood and severity of myocardial injury, thus inducing higher rates of treatment-induced heart failure. 

## 4. Conclusions

In conclusion, despite being a promising adjunctive treatment option for younger female patients affected by breast cancer, GnRH analogues still do not have a fully characterized risk profile, especially when it comes to cardiovascular health. Their potential triggering action for the development of cardiovascular risk factors may induce higher rates of ischemic heart disease in the long run, further exacerbating the already-known cardiotoxic effects of anthracycline-based chemotherapy. Unfortunately, few studies have thoroughly investigated this possibility in women, and tailored randomized control trials with long follow-up are warranted to prove or dismantle evidence of this correlation. In the meanwhile, women treated with GnRH analogues for breast cancer should be involved in a strong primary prevention plan aimed at reducing the incidence of these collateral effects. Encouraging heart-healthy lifestyle changes, such as maintaining a balanced diet, engaging in regular physical activity, and avoiding smoking, is essential for premenopausal women undergoing OFS.

## Data Availability

Not applicable.

## References

[1] Siegel R.L., Miller K.D., Wagle N.S., Jemal A. (2023). Cancer statistics, 2023. CA Cancer J. Clin..

[2] Cancer Statistics Center Breast at a Glance. https://cancerstatisticscenter.cancer.org/?_ga=2.95677403.676965058.1694424103-605706838.1694424103&_gl=1*i5be81*_ga*NjA1NzA2ODM4LjE2OTQ0MjQxMDM.*_ga_12CJLLFFQT*MTY5NDQyNDEwMy4xLjEuMTY5NDQyNDQ2Mi4wLjAuMA.#!/cancer-site/Breast.

[3] Breast Cancer in Young Women. https://www.cdc.gov/cancer/breast/young_women/bringyourbrave/breast_cancer_young_women/index.htm.

[4] Cardoso F., Kyriakides S., Ohno S., Penault-Llorca F., Poortmans P., Rubio I.T., Zackrisson S., Senkus E. (2019). Early breast cancer: ESMO Clinical Practice Guidelines for diagnosis, treatment and follow-up^†^. Ann. Oncol..

[5] (2023). Early and Locally Advanced Breast Cancer: Diagnosis and Management.

[6] Cancer E.T.F.B. (2021). Best Practice Guidelines.

[7] Ortmann O., Weiss J.M., Diedrich K. (2002). Gonadotrophin-releasing hormone (GnRH) and GnRH agonists: Mechanisms of action. Reprod. Biomed. Online.

[8] Huerta-Reyes M., Maya-Núñez G., Pérez-Solis M.A., López-Muñoz E., Guillén N., Olivo-Marin J.C., Aguilar-Rojas A. (2019). Treatment of Breast Cancer with Gonadotropin-Releasing Hormone Analogs. Front. Oncol..

[9] Cheung L.W.T., Wong A.S.T. (2008). Gonadotropin-releasing hormone: GnRH receptor signaling in extrapituitary tissues. FEBS J..

[10] Aguilar-Rojas A., Huerta-Reyes M. (2009). Human gonadotropin-releasing hormone receptor-activated cellular functions and signaling pathways in extra-pituitary tissues and cancer cells (Review). Oncol. Rep..

[11] Sarma P.K.S., Tiwari A., Kondaskar A., Cliffe I.A. (2006). Peptidomimetic GnRH receptor antagonists for the treatment of reproductive and proliferative diseases. Expert. Opin. Ther. Pat..

[12] Millar R.P., Zhu Y.F., Chen C., Struthers R.S. (2000). Progress towards the development of non-peptide orally-active gonadotropin-releasing hormone (GnRH) antagonists: Therapeutic implications. Br. Med. Bull..

[13] Tukun F.L., Olberg D.E., Riss P.J., Haraldsen I., Kaass A., Klaveness J. (2017). Recent Development of Non-Peptide GnRH Antagonists. Molecules.

[14] Francis P.A., Pagani O., Fleming G.F., Walley B.A., Colleoni M., Láng I., Gómez H.L., Tondini C., Ciruelos E., Burstein H.J. (2018). Tailoring Adjuvant Endocrine Therapy for Premenopausal Breast Cancer. N. Engl. J. Med..

[15] LHRH-Agonists in Early Breast Cancer Overview Group (2007). Use of luteinising-hormone-releasing hormone agonists as adjuvant treatment in premenopausal patients with hormone-receptor-positive breast cancer: A meta-analysis of individual patient data from randomised adjuvant trials. Lancet.

[16] Bajo A.M., Schally A.V., Halmos G., Nagy A. (2003). Targeted doxorubicin-containing luteinizing hormone-releasing hormone analogue AN-152 inhibits the growth of doxorubicin-resistant MX-1 human breast cancers. Clin. Cancer Res..

[17] Nagy A., Plonowski A., Schally A.V. (2000). Stability of cytotoxic luteinizing hormone-releasing hormone conjugate (AN-152) containing doxorubicin 14-O-hemiglutarate in mouse and human serum in vitro: Implications for the design of preclinical studies. Proc. Natl. Acad. Sci. USA.

[18] Emons G., Kaufmann M., Gorchev G., Tsekova V., Gründker C., Günthert A.R., Hanker L.C., Velikova M., Sindermann H., Engel J. (2010). Dose escalation and pharmacokinetic study of AEZS-108 (AN-152), an LHRH agonist linked to doxorubicin, in women with LHRH receptor-positive tumors. Gynecol. Oncol..

[19] Lyon A.R., López-Fernández T., Couch L.S., Asteggiano R., Aznar M.C., Bergler-Klein J., Boriani G., Cardinale D., Cordoba R., Cosyns B. (2022). 2022 ESC Guidelines on cardio-oncology developed in collaboration with the European Hematology Association (EHA), the European Society for Therapeutic Radiology and Oncology (ESTRO) and the International Cardio-Oncology Society (IC-OS). Eur. Heart J..

[20] Anagnostis P., Christou K., Artzouchaltzi A.-M., Gkekas N.K., Kosmidou N., Siolos P., Paschou S.A., Potoupnis M., Kenanidis E., Tsiridis E. (2019). Early menopause and premature ovarian insufficiency are associated with increased risk of type 2 diabetes: A systematic review and meta-analysis. Eur. J. Endocrinol..

[21] Margolis K.L., Bonds D.E., Rodabough R.J., Tinker L., Phillips L.S., Allen C., Bassford T., Burke G., Torrens J., Howard B.V. (2004). Effect of oestrogen plus progestin on the incidence of diabetes in postmenopausal women: Results from the Women’s Health Initiative Hormone Trial. Diabetologia.

[22] Kanaya A.M., Grady D., Barrett-Connor E. (2002). Explaining the sex difference in coronary heart disease mortality among patients with type 2 diabetes mellitus: A meta-analysis. Arch. Intern. Med..

[23] Tatulashvili S., Gusto G., Cosson E., Balkau B., Gourdy P., Bonnet F., Bihan H., Fagherazzi G. (2021). Gonadal hormonal factors before menopause and incident type 2 diabetes in women: A 22-year follow-up of 83 799 women from the E3N cohort study. J. Diabetes.

[24] Alonso-Magdalena P., Ropero A.B., Carrera M.P., Cederroth C.R., Baquié M., Gauthier B.R., Nef S., Stefani E., Nadal A. (2008). Pancreatic insulin content regulation by the estrogen receptor ER alpha. PLoS ONE.

[25] Luo J., Manson J.E., Urrutia R.P., Hendryx M., LeBlanc E.S., Margolis K.L. (2017). Risk of Diabetes After Hysterectomy with or Without Oophorectomy in Postmenopausal Women. Am. J. Epidemiol..

[26] Keating N.L., O’Malley A.J., Smith M.R. (2006). Diabetes and cardiovascular disease during androgen deprivation therapy for prostate cancer. J. Clin. Oncol..

[27] Hu J.R., Duncan M.S., Morgans A.K., Brown J.D., Meijers W.C., Freiberg M.S., Salem J.E., Beckman J.A., Moslehi J.J. (2020). Cardiovascular Effects of Androgen Deprivation Therapy in Prostate Cancer: Contemporary Meta-Analyses. Arterioscler. Thromb. Vasc. Biol..

[28] Davidson N.E., O’Neill A.M., Vukov A.M., Osborne C.K., Martino S., White D.R., Abeloff M.D. (2005). Chemoendocrine Therapy for Premenopausal Women with Axillary Lymph Node–Positive, Steroid Hormone Receptor–Positive Breast Cancer: Results from INT 0101 (E5188). J. Clin. Oncol..

[29] Russo M., Della Sala A., Tocchetti C.G., Porporato P.E., Ghigo A. (2021). Metabolic Aspects of Anthracycline Cardiotoxicity. Curr. Treat. Options Oncol..

[30] De Lima Junior E.A., Yamashita A.S., Pimentel G.D., De Sousa L.G., Santos R.V., Gonçalves C.L., Streck E.L., de Lira F.S., Rosa Neto J.C. (2016). Doxorubicin caused severe hyperglycaemia and insulin resistance, mediated by inhibition in AMPk signalling in skeletal muscle. J. Cachexia Sarcopenia Muscle.

[31] Wagenknecht L.E., Zaccaro D., Espeland M.A., Karter A.J., O’Leary D.H., Haffner S.M. (2003). Diabetes and Progression of Carotid Atherosclerosis. Arterioscler. Thromb. Vasc. Biol..

[32] Padro T., Manfrini O., Bugiardini R., Canty J., Cenko E., De Luca G., Duncker D.J., Eringa E.C., Koller A., Tousoulis D. (2020). ESC Working Group on Coronary Pathophysiology and Microcirculation position paper on ‘coronary microvascular dysfunction in cardiovascular disease’. Cardiovasc. Res..

[33] Manfrini O., Cenko E., Bugiardini R. (2020). Gender Differences in Residual Risk Factors for Major Adverse Cardiovascular Events Following ACS and How to Bridge the Gap. Curr. Atheroscler. Rep..

[34] Magkos F., Mittendorfer B. (2009). Gender Differences in Lipid Metabolism and the Effect of Obesity. Obstet. Gynecol. Clin..

[35] Verhoeven M.O., van der Mooren M.J., Teerlink T., Verheijen R.H., Scheffer P.G., Kenemans P. (2009). The influence of physiological and surgical menopause on coronary heart disease risk markers. Menopause.

[36] Matthews K.A., Crawford S.L., Chae C.U., Everson-Rose S.A., Sowers M.F., Sternfeld B., Sutton-Tyrrell K. (2009). Are changes in cardiovascular disease risk factors in midlife women due to chronological aging or to the menopausal transition?. J. Am. Coll. Cardiol..

[37] Derby C.A., Crawford S.L., Pasternak R.C., Sowers M., Sternfeld B., Matthews K.A. (2009). Lipid changes during the menopause transition in relation to age and weight: The Study of Women’s Health Across the Nation. Am. J. Epidemiol..

[38] Hinks T.S.C., Cureton L., Knight R., Wang A., Cane J.L., Barber V.S., Black J., Dutton S.J., Melhorn J., Jabeen M. (2021). Azithromycin versus standard care in patients with mild-to-moderate COVID-19 (ATOMIC2): An open-label, randomised trial. Lancet Respir. Med..

[39] Legro R.S., Kunselman A.R., Dunaif A. (2001). Prevalence and predictors of dyslipidemia in women with polycystic ovary syndrome. Am. J. Med..

[40] Rocha M.P., Maranhão R.C., Seydell T.M., Barcellos C.R., Baracat E.C., Hayashida S.A., Bydlowski S.P., Marcondes J.A. (2010). Metabolism of triglyceride-rich lipoproteins and lipid transfer to high-density lipoprotein in young obese and normal-weight patients with polycystic ovary syndrome. Fertil. Steril..

[41] Bühler K., Winkler U., Schindler A.E. (1992). Influence on hormone levels, lipid metabolism and reversibility of endocrinological changes after leuprorelin acetate depot therapy. Clin. Ther..

[42] Gerhard I., Schindler A.E., Bühler K., Winkler U., Meinen K., Mancarella D., Hoffmann G., Schüssler B., Kimmig R., Kranzfelder D. (1992). Treatment of endometriosis with leuprorelin acetate depot: A German multicentre study. Clin. Ther..

[43] Cheung T.K., Lo K.W., Lam C.W., Lau W., Lam P.K. (2000). A crossover study of triptorelin and leuprorelin acetate. Fertil. Steril..

[44] Al-Omari W.R., Nassir U.N., Sulaiman W.R. (1999). Estrogen and lipid profile in patients with endometriosis treated by GnRH agonist. Int. J. Gynaecol. Obstet..

[45] Howell A., Ashcroft L., Fallowfield L., Eccles D.M., Eeles R.A., Ward A., Brentnall A.R., Dowsett M., Cuzick J.M., Greenhalgh R. (2018). RAZOR: A Phase II Open Randomized Trial of Screening Plus Goserelin and Raloxifene Versus Screening Alone in Premenopausal Women at Increased Risk of Breast Cancer. Cancer Epidemiol. Biomark. Prev..

[46] Palomba S., Orio F., Russo T., Falbo A., Cascella T., Doldo P., Nappi C., Lombardi G., Mastrantonio P., Zullo F. (2004). Long-term effectiveness and safety of GnRH agonist plus raloxifene administration in women with uterine leiomyomas. Hum. Reprod..

[47] Palomba S., Russo T., Orio F., Sammartino A., Sbano F.M., Nappi C., Colao A., Mastrantonio P., Lombardi G., Zullo F. (2004). Lipid, glucose and homocysteine metabolism in women treated with a GnRH agonist with or without raloxifene. Hum. Reprod..

[48] Love R.R., Wiebe D.A., Feyzi J.M., Newcomb P.A., Chappell R.J. (1994). Effects of tamoxifen on cardiovascular risk factors in postmenopausal women after 5 years of treatment. J. Natl. Cancer Inst..

[49] Reckless J., Metcalfe J.C., Grainger D.J. (1997). Tamoxifen Decreases Cholesterol Sevenfold and Abolishes Lipid Lesion Development in Apolipoprotein E Knockout Mice. Circulation.

[50] Klinnikova M.G., Lushnikova E.L., Koldysheva E.V., Tolstikova T.G., Sorokina I.V., Yuzhik E.I., Mzhelskaya M.M. (2016). Cardiotoxic and Dyslipidemic Effects of Doxorubicin and Betulinic Acid Amide. Bull. Exp. Biol. Med..

[51] Sharma H., Pathan R.A., Kumar V., Javed S., Bhandari U. (2011). Anti-apoptotic potential of rosuvastatin pretreatment in murine model of cardiomyopathy. Int. J. Cardiol..

[52] He T., Wang C., Tan Q., Wang Z., Li J., Chen T., Cui K., Wu Y., Sun J., Zheng D. (2020). Adjuvant chemotherapy-associated lipid changes in breast cancer patients: A real-word retrospective analysis. Medicine.

[53] Oka R., Utsumi T., Endo T., Yano M., Kamijima S., Kamiya N., Shirai K., Suzuki H. (2016). Effect of androgen deprivation therapy on arterial stiffness and serum lipid profile changes in patients with prostate cancer: A prospective study of initial 6-month follow-up. Int. J. Clin. Oncol..

[54] Samargandy S., Matthews K.A., Brooks M.M., Barinas-Mitchell E., Magnani J.W., Janssen I., Hollenberg S.M., El Khoudary S.R. (2020). Arterial Stiffness Accelerates Within 1 Year of the Final Menstrual Period. Arterioscler. Thromb. Vasc. Biol..

[55] Harrison-Bernard L.M., Schulman I.H., Raij L. (2003). Postovariectomy Hypertension Is Linked to Increased Renal AT1 Receptor and Salt Sensitivity. Hypertension.

[56] Thomsen M., Nordestgaard B.G. (2014). Myocardial Infarction and Ischemic Heart Disease in Overweight and Obesity with and Without Metabolic Syndrome. JAMA Intern. Med..

[57] Karelis A.D., Faraj M., Bastard J.P., St-Pierre D.H., Brochu M., Prud’homme D., Rabasa-Lhoret R. (2005). The metabolically healthy but obese individual presents a favorable inflammation profile. J. Clin. Endocrinol. Metab..

[58] Fauchier G., Bisson A., Bodin A., Herbert J., Semaan C., Angoulvant D., Ducluzeau P.H., Lip G.Y.H., Fauchier L. (2021). Metabolically healthy obesity and cardiovascular events: A nationwide cohort study. Diabetes Obes. Metab..

[59] Kramer C.K., Zinman B., Retnakaran R. (2013). Are metabolically healthy overweight and obesity benign conditions? A systematic review and meta-analysis. Ann. Intern. Med..

[60] Fantuzzi G., Mazzone T. (2007). Adipose tissue and atherosclerosis: Exploring the connection. Arterioscler. Thromb. Vasc. Biol..

[61] Janssen I., Powell L.H., Jasielec M.S., Kazlauskaite R. (2015). Covariation of change in bioavailable testosterone and adiposity in midlife women. Obesity.

[62] Sella T., Zheng Y., Tan-Wasielewski Z., Rosenberg S.M., Poorvu P.D., Tayob N., Ruddy K.J., Gelber S.I., Tamimi R.M., Schapira L. (2022). Body weight changes and associated predictors in a prospective cohort of young breast cancer survivors. Cancer.

[63] Ricci B., Cenko E., Vasiljevic Z., Stankovic G., Kedev S., Kalpak O., Vavlukis M., Zdravkovic M., Hinic S., Milicic D. (2017). Acute Coronary Syndrome: The Risk to Young Women. J. Am. Heart Assoc..

[64] Cenko E., Yoon J., Kedev S., Stankovic G., Vasiljevic Z., Krljanac G., Kalpak O., Ricci B., Milicic D., Manfrini O. (2018). Sex Differences in Outcomes After STEMI: Effect Modification by Treatment Strategy and Age. JAMA Intern. Med..

[65] Bugiardini R., Manfrini O., Cenko E. (2019). Female sex as a biological variable: A review on younger patients with acute coronary syndrome. Trends Cardiovasc. Med..

[66] Koller A. (2014). Perspectives: Microvascular endothelial dysfunction and gender. Eur. Heart J. Suppl..

[67] Seegers L.M., Araki M., Nakajima A., Yonetsu T., Minami Y., Ako J., Soeda T., Kurihara O., Higuma T., Kimura S. (2022). Sex Differences in Culprit Plaque Characteristics Among Different Age Groups in Patients with Acute Coronary Syndromes. Circ. Cardiovasc. Interv..

[68] Burke A.P., Farb A., Malcom G., Virmani R. (2001). Effect of menopause on plaque morphologic characteristics in coronary atherosclerosis. Am. Heart J..

[69] Pinto S., Coppo M., Bruni V., Rosati D., Cirri R., Abbate R. (1991). Changes in thromboxane A2 generation and plasma lipid pattern in pseudomenopause induced by gonadotropin releasing hormone (GnRH) analogue buserelin. Prostaglandins Leukot. Essent. Fat. Acids.

[70] Muka T., Oliver-Williams C., Colpani V., Kunutsor S., Chowdhury S., Chowdhury R., Kavousi M., Franco O.H. (2016). Association of Vasomotor and Other Menopausal Symptoms with Risk of Cardiovascular Disease: A Systematic Review and Meta-Analysis. PLoS ONE.

[71] El Khoudary S.R., Aggarwal B., Beckie T.M., Hodis H.N., Johnson A.E., Langer R.D., Limacher M.C., Manson J.E., Stefanick M.L., Allison M.A. (2020). Menopause Transition and Cardiovascular Disease Risk: Implications for Timing of Early Prevention: A Scientific Statement from the American Heart Association. Circulation.

[72] Chou Y.S., Wang C.C., Hsu L.F., Chuang P.H., Cheng C.F., Li N.H., Chen C.C., Chen C.L., Lai Y.J., Yen Y.F. (2023). Gonadotropin-releasing hormone agonist treatment and ischemic heart disease among female patients with breast cancer: A cohort study. Cancer Med..

[73] Davies N.G., Jarvis C.I., Edmunds W.J., Jewell N.P., Diaz-Ordaz K., Keogh R.H. (2021). Increased mortality in community-tested cases of SARS-CoV-2 lineage B.1.1.7. Nature.

[74] Francis P.A., Regan M.M., Fleming G.F., Láng I., Ciruelos E., Bellet M., Bonnefoi H.R., Climent M.A., Da Prada G.A., Burstein H.J. (2014). Adjuvant Ovarian Suppression in Premenopausal Breast Cancer. N. Engl. J. Med..

[75] Darby S.C., Ewertz M., McGale P., Bennet A.M., Blom-Goldman U., Brønnum D., Correa C., Cutter D., Gagliardi G., Gigante B. (2013). Risk of ischemic heart disease in women after radiotherapy for breast cancer. N. Engl. J. Med..

[76] Tan C., Tasaka H., Yu K.P., Murphy M.L., Karnofsky D.A. (1967). Daunomycin, an antitumor antibiotic, in the treatment of neoplastic disease. Clinical evaluation with special reference to childhood leukemia. Cancer.

